# MALDI-MSI—A Step Forward in Overcoming the Diagnostic Challenges in Ovarian Tumors

**DOI:** 10.3390/ijerph17207564

**Published:** 2020-10-18

**Authors:** Dagmara Pietkiewicz, Agnieszka Horała, Szymon Plewa, Piotr Jasiński, Ewa Nowak-Markwitz, Zenon J. Kokot, Jan Matysiak

**Affiliations:** 1 Department of Inorganic and Analytical Chemistry, Poznan University of Medical Sciences, 6 Grunwaldzka Street, 60-780 Poznan, Poland; dagmarapietkiewicz3@gmail.com (D.P.); splewa@ump.edu.pl (S.P.); 2 Gynecologic Oncology Department, Poznan University of Medical Sciences, 33 Polna Street, 60-535 Poznan, Poland; ahorala@ump.edu.pl (A.H.); ewamarkwitz@ump.edu.pl (E.N.-M.); 3 Department of Pathology Gynecological and Obstetric Clinical Hospital, Poznan University of Medical Sciences, 33 Polna Street, 60-535 Poznan, Poland; pjasinski@gpsk.ump.edu.pl; 4 Faculty of Health Sciences, Calisia University, 13 Kaszubska Street, 62-800 Kalisz, Poland; z.kokot@akademiakaliska.edu.pl

**Keywords:** proteomics, mass spectrometry, MALDI-MSI, tissue imaging, ovarian tumors

## Abstract

This study presents the use of matrix-assisted laser desorption and ionization mass spectrometry imaging (MALDI-MSI) directly on the tissue of two ovarian tumors that often present a diagnostic challenge, a low-grade serous borderline ovarian tumor and ovarian fibrothecoma. Different spatial distribution of m/z values within the tissue samples was observed, and regiospecific peaks were identified. Among the 106 peaks in the borderline ovarian tumor five, regiospecific peaks (m/z: 2861.35; 2775.79; 3368.34; 3438.43; 4936.37) were selected using FlexImaging software. Subsequently, the distribution of those selected peaks was visualized on the fibrothecoma tissue section, which demonstrated the differences in the tissue homo-/heterogeneous structure of both tumors. The comparison with the histopathological staining of the ovarian borderline tumor tissue section, obtained during serial sectioning, showed a close correlation of the molecular map with the morphological and histopathological features of the tissue and allowed the identification of different tissue types within the sample. This study highlights the potential significance of MSI in enabling morphological characterization of ovarian tumors as well as correct diagnosis and further prognosis than thus far seen in the literature. Osteopontin, tropomyosin and orosomucoid are only a couple of the molecules investigated using MALDI-MSI in ovarian cancer research. This study, in line with the available literature, proves the potential of MALDI-MSI to overcome the current limitations of classic histopathological examination giving a more in-depth insight into the tissue structure and thus lead to the more accurate differential diagnosis of ovarian tumors, especially in the most challenging cases.

## 1. Introduction

Ovarian tumors are a common gynecological health problem. Diagnostic methods currently used in clinical practice in order to detect ovarian tumors encompass a basic gynecological bimanual examination and a transvaginal ultrasound scan. Most of the detected ovarian tumors are benign, and some do not require any treatment. There are several diagnostic tools used to assess the probability of ovarian malignancy, such as two serum biomarkers, cancer antigen 125 (CA125) and human epididymis protein 4 (HE4), ultrasound features, clinical information and combinations of all listed above. However, the sensitivity and specificity of the available diagnostic tests are limited [1]. Therefore, it is often difficult to assess preoperatively if an ovarian tumor is benign or malignant. Most of the patients are qualified for surgical treatment as histopathological examination of the resected ovarian tissue, as this is a golden standard in confirming the diagnosis and so far the only reliable method of differentiating between benign and malignant tumors of the ovary. An intraoperative histopathological examination is also widely used to assess the type of tumor, allowing the surgeon to choose the proper treatment during the actual surgery. This information is crucial for deciding if fertility-sparing treatment can be applied or if radical oncological surgery is needed. Thus, a precise intraoperative tissue examination is often indispensable and has significant clinical consequences.

Ovarian tumors are a heterogeneous group of neoplasms which develop from different kinds of tissue: the epithelium (65–70%), the germ cells (15–20%) and the ovarian stroma (5–10%); or which can be metastatic from other organs (5%). The vast majority of the malignant ovarian tumors (about 90%) are of epithelial origin and can be further divided into the following subtypes: serous, endometrioid, clear cell, mucinous [2]. Various studies have shown that even though these subtypes all originate from the epithelial tissue, they have unique sequences of tumorigenesis. In consequence, ovarian cancer is a highly heterogenic disease composed of a diverse group of tumors that have distinctive morphological and molecular genetic features [3]. 

Borderline ovarian tumors (BOTs), also called atypical proliferative tumors, are a special group of ovarian tumors characterized by atypical cellular proliferation but without stromal invasion [4]. As BOTs can be associated with microinvasion, intraepithelial carcinoma, lymph node involvement or non-invasive peritoneal implants [5], the histopathological distinction of BOTs from malignant tumors can be challenging. Moreover, the diagnostic criteria for the less common histologic subtypes are not clearly defined [6].

Histopathological examination is often complemented by immunohistochemistry (IHC) to identify tumor-specific proteins—a time–consuming method but often essential for establishing the correct diagnosis. Another significant problem in histopathology is tissue heterogeneity. Areas with low tumor differentiation determine prognosis, even if most of the tumor area is well differentiated. Methods used in traditional biomarkers discovery studies (e.g., peptide/protein profiling) that examine blood, serum or even tissue homogenate results in the loss of crucial histological information and significant differences that might be observed only as a localized histological target concentration [7]. 

Due to the above-mentioned considerations, the histopathological examination of ovarian tumor tissue can be very challenging. To overcome the limitations of classic histological imaging methods, in the era of ‘multi-omics’, molecular imaging was proposed as a tool for acquiring spatial and quantitative information about thousands of molecules without labelling potential targets [8]. While tumor tissue reflects all proteomic, metabolomic, and genetic changes, direct investigation of the tissue sample provides comprehensive information about tumor origin and heterogeneity [9] and may also provide prognostic information. Therefore, the matrix-assisted laser desorption and ionization mass spectrometry imaging (MALDI-MSI) technique, which has added to proteomics this anatomical dimension, is rapidly developing and has received significant attention in recent years in the field of biomarker search. This two-dimensional mass spectrometry technique is intended for the visualization of the spatial distribution of the biomolecules within the tissue. It is suited for the study of various types of molecular compounds, with proteins, peptides and lipids being the most commonly investigated. One of the diseases investigated so far was ovarian cancer, where serous ovarian cancer was the most common subject of research It was indicated that some compounds like osteopontin [10], tropomyosin [11], orosomucoid [12] and N-glycans [13,14] have an altered expression and might be correlated with the presence of ovarian cancer or ovarian cancer progression. 

MALDI-MSI allows tissue integrity to be maintained while obtaining information about the spatial distribution of multiple peptide ions objectively [15]. Compared to IHC, the amount of tissue needed for MSI analysis is small, which is a great advantage considering the amount of additional molecular testing performed to ensure the best patient care [16]. The spectral data obtained from the MALDI-MSI experiments are then calculated and provide mapping information about the distribution of the selected molecules in the tissue sections, making it possible to distinguish between different tissue types within the same section. The obtained data is subsequently correlated with the results of the histopathological studies. Because of the possibility of combining the mass spectrometry (MS) results with morphological features, MALDI-MSI could be a helpful tool for fast and objective tumor classification and grading. 

For this pilot study, we selected two ovarian tumors that often present a diagnostic challenge both for the clinicians and for the histopathologists. The presented research aimed to investigate the usefulness of MALDI-MSI as a complementary tool for histopathological examination in diagnosing ovarian tumors. 

## 2. Materials and Methods

### 2.1. Sample Information

The ovarian tumor tissue samples were obtained during surgeries at the Department of Gynecologic Oncology (Poznan University of Medical Sciences, Poznan, Poland), in accordance with the Declaration of Helsinki, after the approval of the study protocol and of the written information for patients by the Local Bioethical Committee of Poznan University of Medical Sciences, Poland (decision no. 139/20). The tumors were diagnosed during standard histopathological examination and additional immunohistochemistry staining by a pathologist, an expert in ovarian tumors as a low-grade serous borderline tumor (also referred to as atypical proliferative serous tumor) and ovarian fibrothecoma.

### 2.2. Sample Preparation

For the MALDI Imaging experiment, a fresh frozen tissue sample was sectioned at a thickness of 10 μm using a cryostat (Leica Biosystems, Wetzlar, Germany) chilled to −20 °C. The sections were transferred onto pre-cooled indium tin oxide (ITO)-coated glass slides (Bruker Daltonics, Bremen, Germany). Subsequently, each section was thaw mounted on the cooled slide until all visible moisture on it disappeared. The slides carrying the tissue sections were transferred into a vacuum desiccator to dry for 30 min. The tissue sections were treated with wash steps of 70% ethanol (EtOH) (twice, 1 min each) and 96% ethanol (1 min). Then, the tissue sections were dehydrated using a vacuum desiccator for 15 min. The peptide calibration standard II (Bruker Daltonics, Bremen, Germany) was manually spotted (1μl) next to the tissue for calibration of the acquisition method. ITO glass slides were marked and scanned at 3200 dpi on a Reflecta MF 5000 scanner for instrument teaching purposes. The α-Cyano-4-hydroxycinnamic acid (HCCA) matrix stock solution (7g/L) in 50% acetonitrile (ACN), 0.2% (v/v) trifluoroacetic acid (TFA) was further diluted with ACN in a volume ratio of 3:1 and then applied onto the prepared glass slide with a tissue section using a method of the ImagePrep station (Bruker Daltonics, Bremen, Germany). Matrix solution application method was optimized to achieve a homogeneous matrix layer and then obtain good quality MS analysis results (Table 1).

### 2.3. MALDI-MSI

MS data were acquired using an UltrafleXtreme MALDI-TOF/TOF instrument (Bruker Daltonics, Bremen, Germany) controlled by flexControl (v3.4, Bruker Daltonics, Bremen, Germany) and flexImaging (v4.1, Bruker Daltonics, Bremen, Germany) in linear positive ion mode. Settings for MALDI-TOF MS analysis were as follows: 2000–20,000 m/z range. To suppress unwanted ions, the matrix suppression parameter was set at 1500Da. Laser parameters were as follows: pulsed ion extraction 260 ns and lens 6.40 kV. 2000 shots were acquired at each position using the Smartbeam 2_small laser diameter within a 200 μm raster width.

### 2.4. Imaging Data Analysis

**Figure 1:**
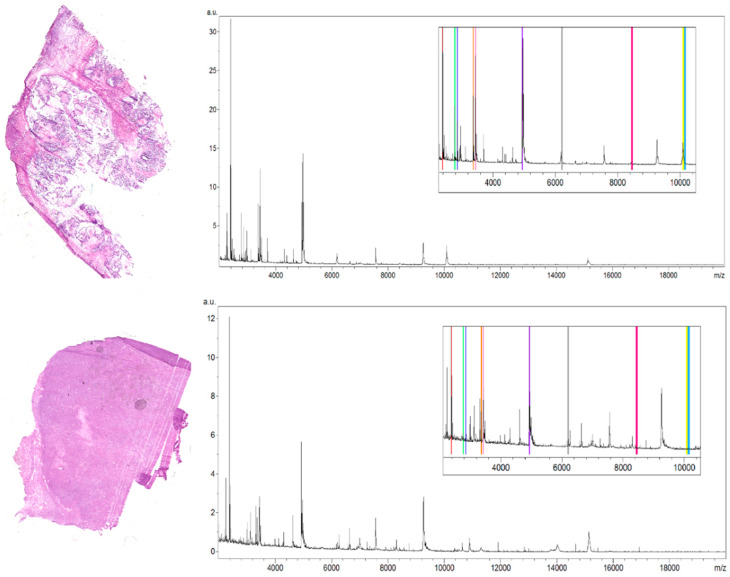
Hematoxylin and eosin stained tissue sections of a low-grade serous borderline ovarian tumor (**top picture**) and ovarian fibrothecoma (**bottom picture**) next to the averaged spectra of the analyzed tissue sections. For each spectrum, the zoomed fragment is presented with color-labeled regiospecific peaks within the borderline tumor tissue section (m/z: 2775.79 (green), 2861.35 (navy blue), 3368.34 (orange), 3438.43 (pink), 4936.37 (purple)) and peaks differentiating these two tissue sections (m/z: 2386.53 (red), 6204.735 (grey), 8453.171 (magenta), 10,146.87 (blue), 10,092.39 (yellow)).

The selection of peaks characterized by the highest regiospecificity in the analyzed ovarian tumor tissue was carried out manually in flexImaging software dedicated to tissue imaging analyses. We defined regions of interest (ROI) for borderline ovarian tumor tissue corresponding to the different tissue subtypes visible on the hematoxylin and eosin (H&E) stained tissue section. Then in the flexImaging we displayed the density plot, and we manually chose the m/z values that seemed to be the most differentiating between ROIs in the borderline ovarian tumor tissue section. The averaged spectra (Figure 1) obtained during analysis of the serous borderline tumor, and the ovarian fibrothecoma, were analyzed, and then, based on the manually selected peaks, the differentiating masses were visualized with the flexImaging software.

### 2.5. Hematoxylin and Eosine Staining

For the hematoxylin and eosin staining, one of the sections obtained during serial sectioning was taken to perform the correlation of MALDI-MSI data with histology. The staining procedure was multi-stage. First, the slide was dip-washed in deionized water. Next, the tissue section was stained in hematoxylin solution for 5 min and dip-washed in deionized water once again to remove an excess of the staining solution. The slide was rinsed in running tap water for 5 min and then washed in deionized water for 1 min. In the next step, the slide was put into eosine solution until the section was sufficiently stained (approximately 1 min) and was washed in deionized water. Then, the tissue sections were washed for two min each, in 70% EtOH, 80% EtOH, 90% EtOH, 96% EtOH, 96% EtOH again and xylene. One droplet of mounting medium was spread using a glass stir bar on the slide, and a coverslip was placed onto the tissue.

## 3. Results

**Figure 2:**
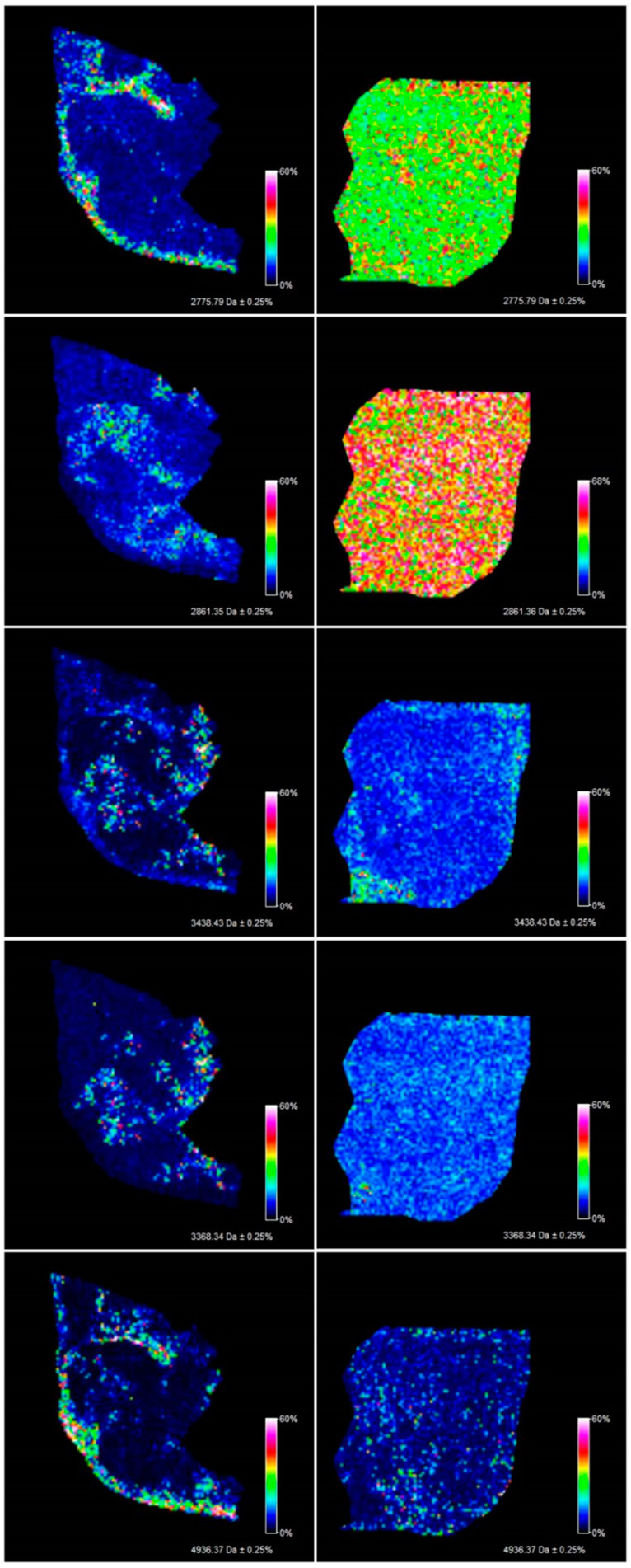
Pictures visualize the homogenous distribution of the regiospecific compounds from the borderline ovarian tumor tissue section (**left**) in the fibrothecoma tissue section (**right**).

**Figure 3:**
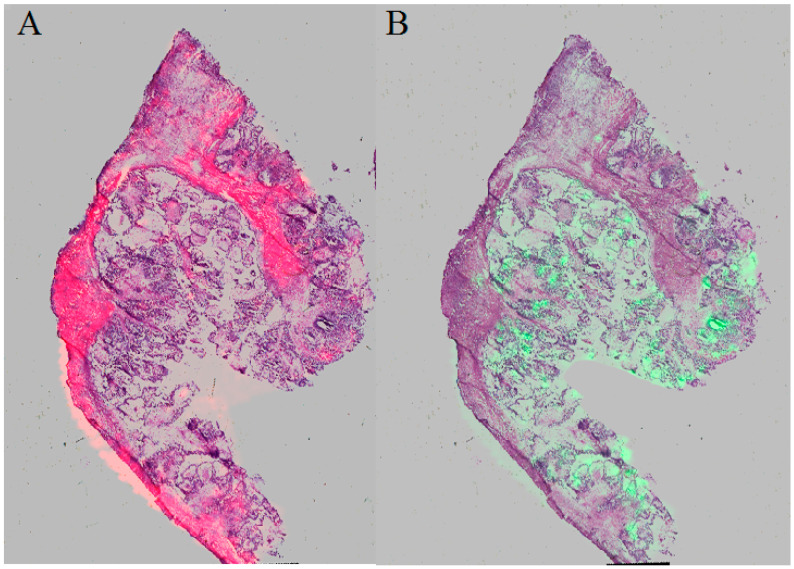
Picture **A** visualizing m/z: 4936.37 and 2775.79 characteristic of mesenchymal tissue and picture **B** visualizing m/z: 2861.35, 3368.34 and 3438.43 characteristic of epithelial tissue.

**Figure 4:**
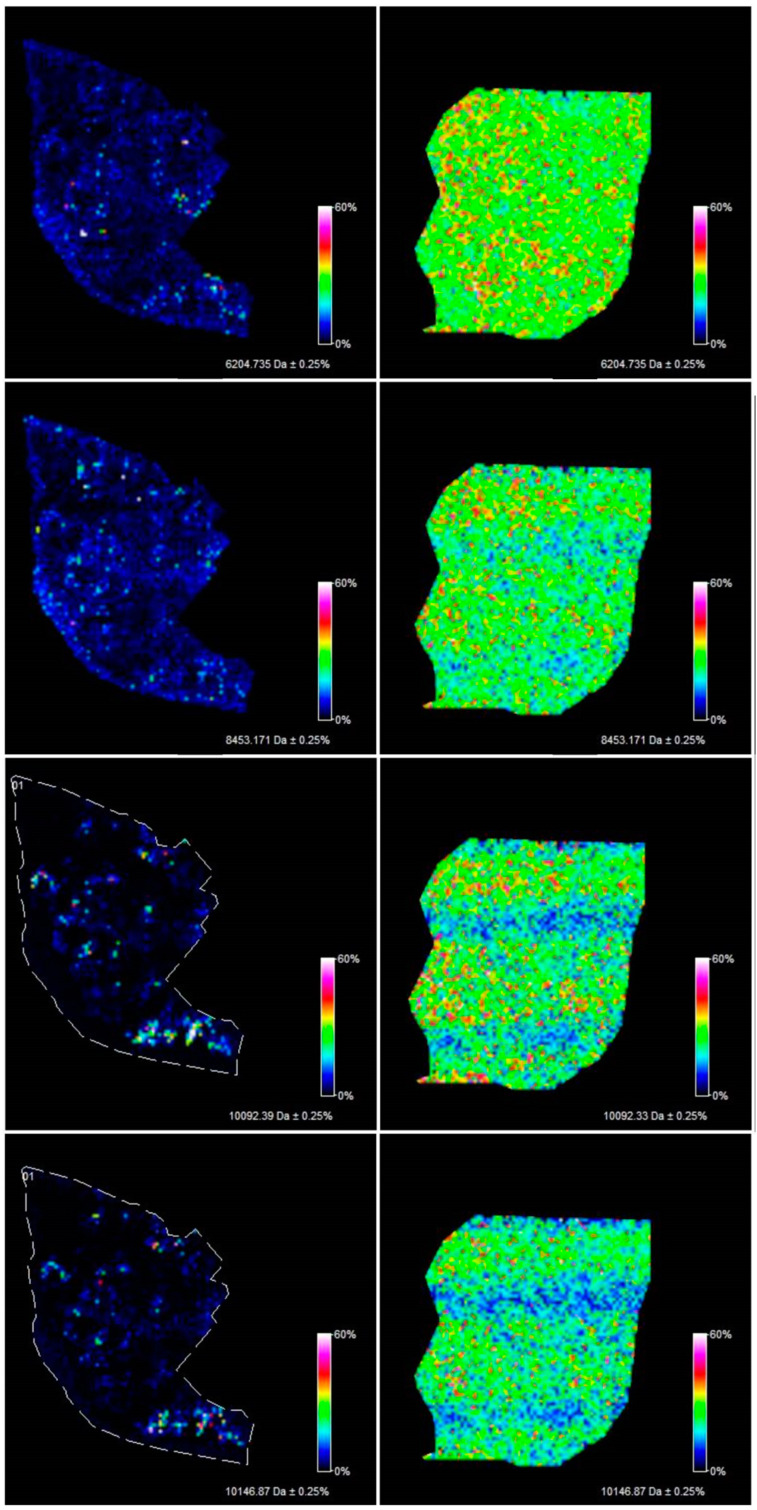
Visualization of the peaks (m/z: 6204.735, 8453.171, 10,146.87, 10,092.39) differentiating the borderline ovarian tissue (**left**) from the ovarian fibrothecoma tissue (**right**) based on the average spectra.

The methodology proposed in this study allowed the measurement of 106 m/z values in the tissue section of the serous borderline tumor and 133 m/z values in the ovarian fibrothecoma tissue section in the 2000–20,000 m/z range (Figure 1). Among the 106 ions from the serous borderline tumor tissue, five regiospecific peaks (m/z: 2861.35; 2775.79; 3368.34; 3438.43; 4936.37) were selected using FlexImaging software and then compared with the distribution of the same masses in the fibrothecoma tissue section. Different distribution of the selected peaks was shown in molecular images (Figure 2). Due to the homogeneous structure of the fibrothecoma tissue, no regiospecific masses were found. Visualization of the regiospecific peaks of the serous borderline tumor on the fibrothecoma tissue section also shows the lack of regiospecific distribution of these masses in the fibrothecoma tissue (Figure 2). The visualization of the differentiated distribution of the selected regiospecific peaks in serous borderline tumor tissue and then the comparison of the results with the histopathological staining of the borderline tumor tissue section, obtained during serial sectioning, allowed the identification of mesenchymal and epithelial tissue within the borderline tumor tissue. The peaks with m/z: 4936.37 and 2775.79 are proposed as characteristic of mesenchymal tissue (Figure 3) and the peaks with m/z: 2861.35, 3368.34 and 3438.43 are proposed as characteristic of epithelial tissue (Figure 3). Epithelial and mesenchymal tissue were assigned based on the microscopic examination of histopathological features in H&E stained sections that were characterized by an expertise pathologist. In addition, the MALDI MSI images were analyzed and discussed with the pathologist, who helped to recognize particular parts of the tissue on the tissue section. In the next step, the serous borderline tumor was compared with the ovarian fibrothecoma. The peaks differentiating both tissues were selected. The peaks at m/z: 2386.53, 6204.735, 8453.171, 10,146.87, 10,092.39 showed the greatest differentiation between the tissues. These m/z values were abundant in the regions of a well-differentiated ovarian tissue whereas in the regions of the borderline tumor with atypical cell proliferation, they were scarce. Differentiating compounds were then visualized and shown on the molecular maps of ovarian fibrothecoma and borderline ovarian tumor (Figure 4).

### Immunohistochemistry (IHC)

**Figure 5:**
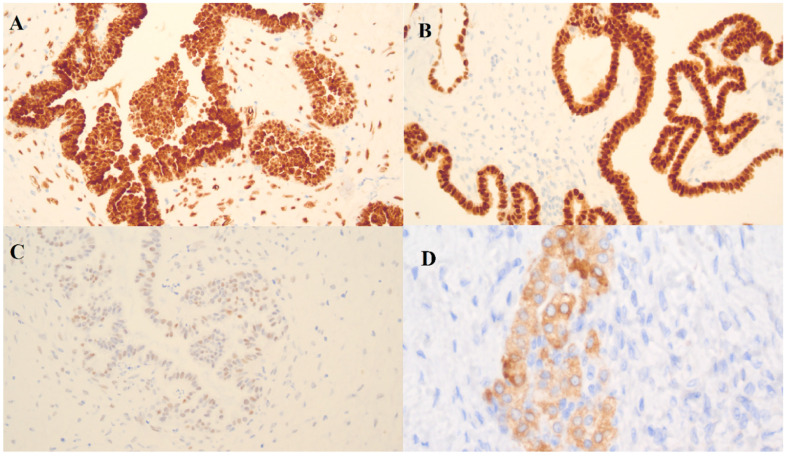
Immunohistochemistry (IHC) detection of (**A**) WT-1, (**B**) PAX-8, (**C**) p53 in a borderline ovarian tumor sample and (**D**) inhibin alpha in an ovarian fibrothecoma sample.

In the borderline ovarian tumor sample, three immunohistochemistry (IHC) markers were used: Wilms’ tumor protein (WT-1), tumor protein p53 (p53) and paired box gene 8 (PAX-8) (Figure 5). There was a positive reaction for WT-1 and PAX-8 and a weak positive reaction for p53. 

The WT-1 gene was originally identified as a tumor suppressor gene and is overexpressed in a variety of neoplasms. WT1 is expressed in the ovarian surface epithelium and stromal and granulosa cells of a healthy ovary [17]. In the tumoral ovary, WT1 is characteristic for the serous subtype of ovarian tumors [17]. PAX-8 is characteristic for the epithelial phenotypes (serous, clear cell and endometrioid) of ovarian tumors and is useful in differentiation between primary ovarian and metastatic tumors of the ovary [17]. The mutated form of p53, a tumor suppressor gene, is indicative of high-grade serous ovarian cancer.

The presence of WT-1 and PAX-8 proteins confirmed the origin of the tumor from the serous epithelium and a weak positive reaction in the few cells for p53 confirms a borderline. 

In the case of the ovarian fibrothecoma, a positive reaction with inhibin alpha was indicative of the steroid activity of the tumor cells and confirmed the diagnosis.

## 4. Discussion

MALDI-MSI has been widely used in research on various diseases (e.g., Parkinson’s disease [18], diabetes [19], Alzheimer’s disease [20]) and the number of its possible applications is constantly growing. Among its potential applications, the analysis of cancer tissue is highly ranked. Cancerous tissue can be analyzed in terms of diagnostics, i.e., differentiation of cancerous tissue from non-cancerous tissue and prognosis, i.e., the ability to predict the disease outcomes. The current use of tissue imaging techniques focuses on the possibility of transferring mass spectrometry to clinical practice, especially when tissue information can be obtained during surgery. Multiple studies have shown that the MSI technique can be used for rapid diagnosis or prognostic testing using patient tissues [21,22,23]. Most of these studies were carried out ex vivo performing the MSI analysis of tissue sections. However, some in vivo studies using alternative technologies, such as the iKnife, are also available [24]. The results obtained in this work, as well as those presented in the available literature, indicate that the future of clinical diagnostics will be strongly influenced by “omic” techniques as a basis for the detection of potential tissue biomarkers. Unlike antibody-based methods, MSI provides a unique opportunity to detect differentiating compounds or other biomolecules found in tissue without knowing the structure of these molecules in advance.

For the purpose of this study, the tissue samples of a low-grade serous borderline ovarian tumor and ovarian fibrothecoma were selected as examples of ovarian lesions whose correct diagnosis is challenging and which might be misdiagnosed as ovarian cancer.

BOTs are a heterogeneous group of ovarian neoplasms characterized by atypical cell proliferation but no destructive invasion of the stroma. Their correct diagnosis can be challenging as the transformation from a benign cystadenoma or cystadenofibroma through BOT into the low-grade serous ovarian cancer is gradual, and it can be hard to draw the line between one and another. According to the World Health Organization (WHO) 2014 classification, tumors with more than 10% of borderline histology within a cystadenoma or cystadenofibroma qualifies as BOT. In contrast, serous cystadenomas with borderline foci that occupy less than 10% of the epithelial volume are referred to as “cystadenoma/fibroma with focal epithelial proliferation”. In addition, these tumors are difficult to differentiate from truly invasive ovarian cancer due to their microscopic features like microinvasion and non-destructive omental implants [25,26].

Although occasionally BOTs may give implants in the omentum and peritoneum, they are considered a distinct clinical entity than ovarian cancer. On the other hand, BOTs share the staging system with invasive ovarian cancer and carry a significant risk of recurrence after conservative surgery. The risk of progression to invasive ovarian cancer seems significant and reached up to 4% in some reports [27]. BOTs generally have an excellent prognosis with a 10-year survival rate of 97% for all stages combined [6], although, as mentioned before, recurrences and malignant transformation can occur. The standard treatment includes complete surgical staging (i.e., hysterectomy with bilateral salpingo-oophorectomy, omentectomy, peritoneal biopsies, cytology of peritoneal washings and appendectomy in case of mucinous BOT), similarly to early-stage ovarian cancer, but adjuvant chemotherapy is not necessary. A large multicenter study revealed that overdiagnosis of a borderline tumor was made in 11.5% (92/803) of patients [28]. As explained above, the diagnosis of BOT has severe clinical implications, and thus its accurate identification is of crucial importance.

Fibrothecomas are rare ovarian neoplasms originating from the sex cord-stroma. They are often found accidentally during a transvaginal ultrasound, magnetic resonance or computer tomography. Large tumors are often associated with pleural effusion and ascites symptoms typically suggestive of advanced-stage ovarian cancer. Diagnosing ovarian fibrothecomas preoperatively can be difficult also due to their solid nature and elevated serum CA-125 levels [29].

In our study, both patients were qualified for surgery due to being suspected of ovarian cancer. The correct diagnosis was only possible after the histopathological examination of the excised lesions complemented by immunohistochemical tests. However, as explained before, these techniques have their limitations. This emphasizes the need to search for a different diagnostic method that could potentially characterize various ovarian neoplasms more objectively, with higher specificity and sensitivity. Our results suggest that MALDI-MSI is a promising tool in identifying biomarkers or biomarker tissue patterns indicative of specific ovarian tumors as well as a specific tissue’s regions. In the literature, there are several articles aimed at either correct ovarian tumor identification on MALDI-MSI or searching for biomarkers. Most of them, however, are focused on ovarian cancer rather than benign ovarian tumors. In the study by Ayed et al. [12], the researchers noticed, similarly to our findings, significant differences between the analyzed tissues and were able to identify putative ovarian cancer biomarkers by the complementary use of liquid chromatography coupled with mass spectrometry of the digested cancerous tissue. Nevertheless, this study compared benign ovarian tumors with advanced-stage ovarian cancer and did not take into account early-stage ovarian cancer, whose diagnosis is the most challenging. Another interesting study in the field concentrated on the correct classification of ovarian cancer histotypes (serous/endometrioid/mucinous/clear cell) and additionally included BOTs arguing that the differential diagnosis between BOT and truly invasive carcinoma can be very challenging (which was also the rationale to choose this particular tissue for our research) [30]. In this study, proteomic information from MALDI-MSI was combined with machine learning approaches to create a model discriminating between different epithelial ovarian cancer subtypes. Although the methods used in both studies were similar, it is impossible to compare the results with our research due to differences in the study design and data processing. Other available studies have rather been focused on the investigation of the distribution of specific markers in the ovarian cancer tissue [13,31]. In the literature search, we did not find any previous studies on the MALDI-MSI used in diagnosing benign ovarian tumors.

In this pilot study, we were able to demonstrate some differentiation not only between ovarian borderline tumor and fibrothecoma but also within the ovarian borderline tumor tissue itself, identifying the regions with increased cell proliferation and the regions of different tissue origin. Moreover, the differences in the tissue homo-/heterogeneous structure of both tumors were shown. Further research could define characteristic m/z peak patterns for each of the ovarian tumors that would allow quick and reliable diagnosis of the ovarian lesion. If, with the advancements in the technique, the time of the MALDI-MSI examination is reduced, it could also be possible to apply it intraoperatively to assist the surgeons. MALDI-MSI could be used to correctly identify if the ovarian lesion is benign, borderline or malignant, to identify its histological subtype (important as, e.g., in mucinous borderline/malignant tumors of the ovary, appendectomy is recommended) or to define the optimal surgical margins.

The results obtained in this pilot study suggest that the MALDI-MSI method is not only complementary to histopathology but might be a technological step forward in the precise diagnosis of ovarian tumors. In addition, MALDI-MSI provides comprehensive information combining current histopathological knowledge with molecular information obtained with the cutting-edge imaging methods, and by identification of the compounds representing specific m/z peaks it could contribute to tissue biomarker discoveries. MSI enables simultaneous, label-free imaging of 100’s–1000’s of compounds directly from the tissue section. Imaging diverse classes of biomolecules (e.g., metabolites, lipids, glycans) gives the opportunity to develop diagnostic/prognostic assays that complement established histopathological and molecular methods. The MALDI-MSI method provides comprehensive visualization of the spatial distribution of potential differentiating compounds between ovarian tumors, which significantly improves the possibilities of biomarker analysis. Performing histological staining after MSI data acquisition enables seamless integration of the molecular information provided by MSI with established histopathological methods [32]. This approach is likely to be used in clinical studies from bench to bedside and may eventually lead to a new quality of diagnosis of ovarian tumors.

While most MALDI-MSI studies investigated ovarian cancer tissue, the distinctive feature of our research is the application of MALDI-MSI technique for the characterization of benign ovarian tumors. We examined two rare ovarian neoplasms, that have not been characterized in the previous studies, but which might often be misdiagnosed as ovarian cancer. As a pilot study, it was restricted to only two samples and naturally, the findings require verification in larger patient cohorts to estimate the robustness of our observations. In the next step, the identification of the selected m/z values should be performed to understand the findings better and to analyze them along with the tumor biology. This study shows the complementarity of the MALDI-MSI technique to the methods used so far in clinical practice, e.g., H&E staining and the results of the performed analyses are promising in identifying biomarkers of specific ovarian tumors or specific tissue regions. Addressing the above-mentioned limitations can be a starting point for future research.

## 5. Conclusions

The MALDI-MSI technique enabled the creation of a molecular map of the spatial distribution of the m/z peaks representing peptides/proteins in the borderline ovarian tumor and ovarian fibrothecoma tissues, both of which may pose a diagnostic challenge for clinicians and for histopathologists. The analysis of the ovarian tumor tissue using MALDI-MSI showed a close correlation of the molecular map with its morphological and histopathological features and allowed us to identify different tissue types within the borderline ovarian tumor tissue section. Moreover, creating molecular maps has shown the differences in the distribution of regiospecific m/z values within the examined samples, which could be useful for a quick and accurate intraoperative diagnosis of defining resection margins. The presented technique may open the door for overcoming the current limitations of classic histological imaging methods, thus leading to the more accurate differential diagnosis of the most challenging cases. This pilot study gives grounds for further research on the application of MALDI-MSI in the differential diagnosis of ovarian tumors.

## Figures and Tables

**Table 1:** ImagePrep parameters of the optimized matrix solution application method.

ImagePrep Parameter	Value
Matrix thickness	0.5 V
Incubation time	30 s
Wetness	40%
Spray power	10%
Spray modulation	30%

## References

[1] Ebell M.H., Culp M., Lastinger K., Dasigi T. (2015). A Systematic Review of the Bimanual Examination as a Test for Ovarian Cancer. Am. J. Prev. Med..

[2] Kroeger P.T., Drapkin R. (2017). Pathogenesis and heterogeneity of ovarian cancer. Curr. Opin. Obstet. Gynecol..

[3] Manuscript A., Origin T. (2011). The Origin and Pathogenesis of Epithelial Ovarian Cancer—A Proposed Unifying Theory. Am. J. Surg. Pathol..

[4] Silverberg S.G., Bell D.A., Kurman R.J., Seidman J.D., Prat J., Ath F., Ronnett B.M., Copeland L., Silva E., Gorstein F. (2004). Borderline Ovarian Tumors: Key Points and Workshop Summary. Hum. Pathol..

[5] Seidman J.D., Kurman R.J. (2000). Ovarian Serous Borderline Tumors:A Critical Review of the Literature With Emphasis on Prognostic Indicators. Hum. Pathol..

[6] Hauptmann S., Friedrich K., Redline R., Avril S. (2017). Ovarian borderline tumors in the 2014 WHO classification: Evolving concepts and diagnostic criteria. Virchows Archiv..

[7] Scott A.J., Jones J.W., Orschell C.M., Macvittie T.J., Kane M.A., Ernst R.K. (2014). Mass spectrometry imaging enriches biomarker discovery approaches with Candidate mapping. Health Phys..

[8] Imaging P., Classic F., Mass M., Histology S.M. (2019). Proteome Imaging: From Classic to Modern Mass Spectrometry-Based Molecular Histology.

[9] Schöne C., Höfler H., Walch A. (2013). MALDI imaging mass spectrometry in cancer research: Combining proteomic profiling and histological evaluation. Clin. Biochem..

[10] Longuespée R., Gagnon H. (2013). Proteomic analyses of serous and endometrioid epithelial ovarian cancers: Cases studies: Molecular insights of a possible histological etiology of serous ovarian cancer. Proteom.-Clin. Appl..

[11] Schwamborn K. (2018). Discerning the Primary Carcinoma in Malignant Peritoneal and Pleural Effusions Using Imaging Mass Spectrometry—A Feasibility Study. Proteom.-Clin. Appl..

[12] El Ayed M. (2010). MALDI imaging mass spectrometry in ovarian cancer for tracking, identifying, and validating biomarkers. Med. Sci. Monit..

[13] Briggs M.T., Condina M.R., Ho Y.Y., Everest-Dass A.V., Mittal P., Kaur G., Oehler M.K., Packer N.H., Hoffmann P. (2019). MALDI Mass Spectrometry Imaging of Early- and Late-Stage Serous Ovarian Cancer Tissue Reveals Stage-Specific N-Glycans. Proteomics.

[14] Everest-Dass A.V., Briggs M.T., Kaur G., Oehler M.K. (2016). N-glycan MALDI Imaging Mass Spectrometry on Formalin-Fixed Paraffin-Embedded Tissue Enables the Delineation of Ovarian Cancer Tissues. Mol. Cell. Proteom..

[15] Yang F.Y., Saqib H.S.A., Ruan Q.Q., You M.S. (2019). Mass spectrometry imaging: An emerging technology for the analysis of metabolites in insects. Arch. Insect Biochem. Physiol..

[16] Aichler M., Walch A. (2015). MALDI Imaging mass spectrometry: Current frontiers and perspectives in pathology research and practice. Lab. Investig..

[17] Iliac L.U.L., Arcangiu M.A.L.U.C., Anevari S.I.C., Anciu M.D., Nofriescu M.O., Linei C.O.A.M.Ă. (2013). The value of PAX8 and WT1 molecules in ovarian cancer diagnosis. Rom. J. Morphol. Embryol..

[18] Hulme H., Fridjonsdottir E., Gunnarsdottir H., Vallianatou T., Zhang X., Wadensten H., Shariatgorji R., Nilsson A., Bezard E., Svenningsson P. (2020). Simultaneous mass spectrometry imaging of multiple neuropeptides in the brain and alterations induced by experimental parkinsonism and L-DOPA therapy. Neurobiol. Dis..

[19] Bunney P., Zink A., Holm A., Billington C., Kotz C. (2017). Imaging mass spectrometry reveals direct albumin fragmentation within the diabetic kidney. Physiol. Behav..

[20] Kaya I., Zetterberg H., Blennow K., Hanrieder J. (2018). Shedding Light on the Molecular Pathology of Amyloid Plaques in Transgenic Alzheimer’s Disease Mice Using Multimodal MALDI Imaging Mass Spectrometry. ACS Chem. Neurosci..

[21] Addie R.D., Ballu B., Bove J.V.M.G., Morreau H. (2015). Current State and Future Challenges of Mass Spectrometry Imaging for Clinical Research. Anal. Chem..

[22] Basu S.S., Regan M.S., Randall E.C., Abdelmoula W.M., Clark A.R., Gimenez-Cassina Lopez B., Cornett D.S., Haase A., Santagata S., Agar N.Y.R. (2019). Rapid MALDI mass spectrometry imaging for surgical pathology. NPJ Precis. Oncol..

[23] Brégeon F., Brioude G., De Dominicis F., Atieh T., D’Journo X.B., Flaudrops C., Rolain J.M., Raoult D., Thomas P.A. (2014). MALDI-ToF mass spectrometry for the rapid diagnosis of cancerous lung nodules. PLoS ONE.

[24] Balog J., Sasi-szabó L., Kinross J., Lewis M.R., Muirhead L.J., Veselkov K., Mirnezami R., Dezs B., Damjanovich L., Darzi A. (2013). Intraoperative Tissue Identification Using Rapid Evaporative Ionisation Mass Spectrometry. Sci. Transl. Med..

[25] Seong S.J., Kim D.H., Kim M.K., Song T. (2015). Controversies in borderline ovarian tumors. J. Gynecol. Oncol..

[26] Sherman M.E., Berman J., Birrer M.J., Cho K.R., Ellenson L.H., Gorstein F., Seidman J.D. (2004). Current challenges and opportunities for research on borderline ovarian tumors. Hum. Pathol..

[27] Vang R., G Hannibal C., Junge J., Frederiksen K., K Kjaer S., Robert J.K. (2018). Long-term Behavior of Serous Borderline Tumors Subdivided Into Atypical Proliferative Tumors and Noninvasive Low-grade Carcinomas: A Population-based Clinicopathologic Study of 942 Cases. Am. J. Surg. Pathol..

[28] Ewald-riegler N., De Gregorio N., Reuss A., Mahner S., Fotopoulou C., Kommoss F., Schmalfeldt B., Hilpert F., Fehm T., Burges A. (2013). Borderline tumours of the ovary: A cohort study of the Arbeitsgmeinschaft Gynäkologische Onkologie (AGO) Study Group. Eur. J. Cancer.

[29] Shen Y., Liang Y., Cheng X., Lu W., Xie X., Wan X. (2018). Ovarian fibroma/fibrothecoma with elevated serum CA125 level: A cohort of 66 cases. Medicine.

[30] Klein O. (2018). MALDI-Imaging for classification of epithelial ovarian cancer histotypes from a tissue microarray using machine learning methods. Proteom.-Clin. Appl..

[31] Hernandez Y.C., Boskamp T., Casadonte R., Hauberg-Lotte L., Oetjen J., Lachmund D., Peter A., Trede D., Kriegsmann K., Kriegsmann M. (2012). Targeted feature extraction in MALDI mass spectrometry imaging to discriminate proteomic profiles of breast and ovarian cancer. Proteom.- Clin. Appl..

[32] Walch A., Rauser S., Deininger S.-O., Höfler H. (2008). MALDI imaging mass spectrometry for direct tissue analysis: A new frontier for molecular histology. Histochem. Cell Biol..

